# Fast oscillatory activity induced by kainate receptor activation in the rat basolateral amygdala *in vitro*

**DOI:** 10.1111/j.1460-9568.2010.07582.x

**Published:** 2011-03

**Authors:** Fiona E Randall, Miles A Whittington, Mark O Cunningham

**Affiliations:** 1 Institute of Neuroscience, Newcastle University, Framlington PlaceNewcastle upon Tyne, UK; 2 Brain Mechanisms for Behaviour Unit, Okinawa Institute of Science and TechnologyOnna-son, Kunigami-gun, Okinawa, Japan

**Keywords:** BLA, gamma oscillations, gap junction, glutamate

## Abstract

The basolateral amygdala (BLA) has a fundamental role in affective processing. *In vivo* studies have revealed rhythmic population activity of a similar type to that seen in the hippocampus and cortical areas during learning tasks. The amygdala contains densely interconnected networks of inhibitory interneurons similar to those responsible for fast network activity generation in the hippocampus and other cortical structures. Here we report that neuronal networks of the BLA in isolation generate persistent, gamma frequency (30–80 Hz) oscillations upon kainate receptor activation with kainic acid. We show that, like other cortical structures, BLA oscillations are completely dependent upon γ-aminobutyric acid (GABA)ergic inhibition. GABA_A_ receptor blockade abolished all oscillations, and the activity was also sensitive to the barbiturate, pentobarbital. Blockade of α-amino-3-hydroxy-5-methyl-4-isoxazolepropionic acid (AMPA) receptors or *N*-methyl-d-aspartate (NMDA) receptors had no significant effect on gamma activity. However, the GluR5-containing kainate receptor-specific antagonist (*S*)-1-(2-amino-2-carboxyethyl)-3-(2-carboxybenzyl) pyrimidine-2,4-dione (UBP302) abolished oscillations–evidence that glutamatergic receptor involvement is predominantly kainate receptor mediated. The mixed AMPA/kainate receptor antagonist 6-nitro-7-sulphamoylbenzo[f]quinoxalone-2,3-dione disodium (NBQX) abolished all oscillatory activity in 8/14 of slices tested. In the remaining slices, gamma frequency activity was abolished to reveal a low-amplitude, NMDA receptor-dependent, beta frequency (10–20 Hz) oscillation. Gamma oscillations are abolished by gap junction blockade. While these data show the BLA capable of generating gamma rhythms in common with other cortical areas studied to date, the network mechanisms appear to be different, suggesting a unique network structure underlies amygdala rhythmogenesis. Understanding how BLA networks produce synchronous activity is paramount to understanding how the BLA executes influence on important cognitive processes such as emotional learning.

## Introduction

Gamma oscillations (30–80 Hz) are observed in the brain in response to sensory stimuli, and have a role in memory formation, processing and retrieval (Buzsaki, 1989; Fell *et al.*, 2001; Kaiser *et al.*, 2003). In structures relevant for these observations (hippocampus, entorhinal cortex and neocortex), gamma rhythms are generated by heterogeneous networks of excitatory principal cells and local circuit interneurons (Whittington *et al.*, 2000; Cunningham *et al.*, 2003, 2004b). Local network gamma frequency is controlled by the kinetics of γ-aminobutyric acid (GABA)_A_ receptor-mediated inhibition (Whittington *et al.*, 1995), which appears to organise local principal cell firing into ensembles recurring with periodicity corresponding to the gamma rhythm (Harris *et al.*, 2003).

The basolateral amygdala (BLA) has long been implicated functionally in emotional behaviour and formation of emotional memories (Kluver & Bucy, 1937; Weiskrantz, 1956; Cahill, 2000; McGaugh, 2000). It has reciprocal connections to areas that generate gamma rhythms during declarative memory encoding, storage and retrieval: rhinal cortices, parahippocampal cortex and hippocampus. Sensory and memory function-related inputs to the BLA are mostly glutamatergic, originating in cortical and thalamic structures (Sah *et al.*, 2003). The BLA consists of three nuclei; lateral (LA), basolateral (BA) and basomedial (Sah *et al.*, 2003). Oscillatory activity in the BLA is thought to have an important role in the consolidation of emotional memories (Pelletier & Pare, 2004). Anatomical studies show BLA networks are similar to those in other oscillating regions, although the amygdala lacks defined laminar structure (McDonald & Mascagni, 2004; Muller *et al.*, 2005; Pantazopoulos *et al.*, 2006). Cells in the rodent LA have intrinsic membrane potential oscillations at theta frequency (Pape *et al.*, 2005). A repertoire of oscillatory frequencies has been recorded in the amygdala *in vivo*, for example slow-wave and delta (Pare & Gaudreau, 1996; Collins *et al.*, 2001), theta (Pare & Gaudreau, 1996; Collins *et al.*, 2001; Seidenbecher *et al.*, 2003; Pape *et al.*, 2005) and gamma (Pagano & Gault, 1964; Collins *et al.*, 2001; Hakami *et al.*, 2009; Levesque *et al.*, 2009; Popescu *et al.*, 2009). These frequencies correspond to those recorded in other oscillating areas, i.e. BLA target areas. Gamma oscillations were observed in the BLA, superimposed on theta rhythms in anaesthetised cats (Collins *et al.*, 2001) and during learning in synchrony with rhinal cortices (Bauer *et al.*, 2007). BLA-mediated facilitation of rhinal interactions could contribute to emotional facilitation of memory formation by the BLA (Paz *et al.*, 2006; Bauer *et al.*, 2007). Recent studies have reported synchronous gamma activity between the BLA and striatum during motor learning tasks (Popescu *et al.*, 2009) and amygdalo-hippocampal formation during seizures (Levesque *et al.*, 2009).

Despite the surge in recent literature linking BLA rhythms to function *in vivo,* to our knowledge only one *in vitro* investigation has been reported. Theta frequency oscillations in response to kainate receptor activation were shown to be gap junction dependent in the rodent LA (Sinfield & Collins, 2006). These authors also showed that gamma oscillations induced by metabotropic glutamate receptor activation were not blocked by gap junction blockade. The oscillations observed by these authors were also much smaller in amplitude than those we report here, possibly explained by the differences in slice preparation methods.

Here we carry out a pharmacological study to show that kainate receptor activation across the BLA in isolation leads to generation of persistent, fast, oscillatory activity similar, but not identical, to that in other oscillating areas.

## Materials and methods

### Slice preparation

All animal procedures were carried out in accordance with the UK Animals (Scientific Procedures) Act 1986. Adult male Wistar rats (150–200 g; Charles River, UK) were anaesthetised with inhaled isofluorane, and then injected with a lethal dose of ketamine (> 100 mg/kg) and xylazine (> 10 mg/kg). Once all reflexes were absent, animals were intracardially perfused with 60 mL modified artificial cerebrospinal fluid (aCSF) made up of (in mm): sucrose, 252; KCl, 3; NaH_2_PO_4_, 1.25; NaHCO_3_, 24; MgSO_4_, 1.25; CaCl_2_, 1.6; glucose, 10. Following brain removal, 450-μm coronal brain slices containing the BLA were cut using a vibratome (Leica Microsystems), and trimmed to leave BLA and the surrounding cortex. No slices with any hippocampus were used to remove the possibility of volume conduction of hippocampal network activity. Slices were held in a holding chamber at room temperature for approximately 1 h (in aCSF used to maintain slices in the interface and holding chambers replacing the sucrose content with 126 mm NaCl, and the MgSO_4_ and CaCl_2_ content of 1.25 mm and 1.6 mm, respectively). The slices were transferred to an interface-chamber for electrophysiological recording, where conditions were maintained at approximately 34 °C at the interface between oxygenated aCSF and humidified 95% O_2_ and 5% CO_2_. No recordings were made until slices had been left to equilibrate for at least 1 h.

### Drugs

All drugs were applied by bath perfusion at concentrations stated in the text. The drugs used were kainic acid (KA; Sigma, Gillingham, UK), (*RS*)-2-amino-3-(3-hydroxy-5-*tert*-butylisoxazol-4-yl) (ATPA; Tocris, Bristol, UK), D-2-amino-5-phosphonovalerate (D-APV; Tocris, Bristol, UK); (±)-4-(4-aminophenyl)-1,2-dihydro-1-methyl-2-propylcarbamoyl-6,7-methylenedioxyphthalazine (SYM 2206; Tocris, Bristol, UK), 6-nitro-7-sulphamoylbenzo[f]quinoxalone-2,3-dione disodium (NBQX; Tocris, Bristol, UK), gabazine (Tocris, Bristol, UK); (*S*)-1-(2-amino-2-carboxyethyl)-3-(2-carboxybenzyl) pyrimidine-2,4-dione (UBP302; Tocris, Bristol, UK); L-glutamic acid (Sigma, Gillingham, UK), carbenoxolone (Sigma, Gillingham, UK), octanol (Fluka, Gillingham, UK), pentobarbital (Sigma, Gillingham, UK) and quinine (Sigma, Gillingham, UK).

### Recording, data acquisition and analysis

Field electrodes were positioned in the BLA about 0.5 mm from the external capsule and 0.5 mm from the cortex, in the region of the BA for experiments other than those with a roaming electrode (see Supporting Information Fig. S1). KA (200–400 nm) was bath-applied in the circulating aCSF. Extracellular field recordings were made using glass microelectrodes pulled to a resistance of 2–5 MΩ filled with aCSF. Intracellular recordings were made using glass microelectrodes pulled to a resistance of 80–100 MΩ and filled with 1 m potassium acetate. For reconstructions of recorded neuron morphology, 2% biocytin was included in the recording microelectrode. After the completion of recording, the slices were fixed in 4% paraformaldehyde in 0.1 m phosphate buffer. Standard avidin-biotin-horseradish peroxidase reaction with diaminobenzidine was used to visualise biocytin-filled neurons.

Data were recorded using an Axoprobe-1A amplifier (Axon Instruments, USA), and recorded on computer via an ITC-16 interface (Instrutech, USA). Data were collected and analysed using Axograph software (Axon Instruments, USA). Fourier transform analysis was used to detect the peak frequencies of the oscillations for 60-s epochs of field data. The peak of this was used as a measure of the peak amplitude of the oscillation, and the area underneath the curve (between 20–80 Hz) as a measure of the area power. For analysis of pharmacological effects on the oscillations, pooled data were used. The peak amplitude and area power of the oscillation for 60-s epochs of recorded oscillations were collected. Statistical analysis of filtered data was performed using Sigmastat 2.03 software (Systat Software, USA). This software determined whether data were parametric or non-parametric before statistical tests were applied. Parametric data from one slice or cell were analysed using a standard paired Student’s *t*-test to measure differences before and after pharmacological manipulation, and results are significantly different if *P* < 0.05. Parametric data were presented as mean ± SEM. For non-parametric data sets, the Mann–Whitney *U*-test was employed. This test compares the medians of two groups of data, and results are statistically significant if *P* < 0.05.

For BLA oscillations the peak amplitude and frequency were used, as the lacking laminar structure gave rise to variable levels of coherence (depicted in area power) that were likely due to cellular composition at an exact electrode position within the BLA network. For inhibitory postsynaptic potential (IPSP) decay time analysis during gamma oscillations, 50 IPSPs were measured during kainate oscillations. IPSP amplitude was determined from the onset to peak of the IPSP, and decay time was measured from the peak to the point of IPSP decay slope at 30% of peak amplitude. To measure IPSP changes in the presence of pentobarbital, 40 IPSPs in control and pentobarbital conditions were measured from three separate principal cells and data pooled for statistical analysis with a standard paired *t*-test.

## Results

### BLA networks can generate gamma oscillations in vitro

**FIG. 1:**
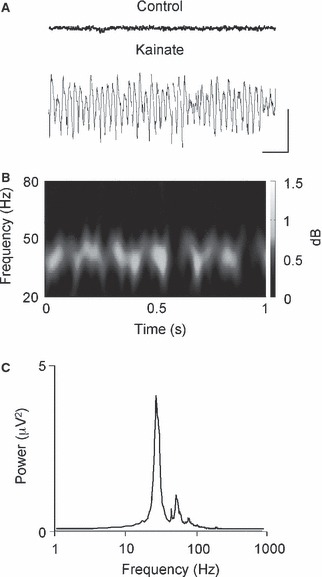
In the presence of KA (200–400 nm), oscillatory activity of gamma frequency builds up in the BLA. (A) Sample trace of local field potential activity recorded extracellularly in the BLA in control aCSF and after the application of kainate to the aCSF. (B) Spectrogram produced using 60-s epochs of gamma activity to illustrate stable frequency of BLA gamma oscillation. (C) Pooled power spectra corresponding to traces in control (grey) and in the presence of kainate (black; *n* = 6). Scale bars: 250 μV, 100 ms (A).

Slices containing BLA did not generate spontaneous rhythmic field potential activity, but bath-application of KA (200–400 nm) led to generation of gamma frequency oscillations. Recording position was determined in slices using the rat brain atlas (Paxinos & Watson, 1998), and the spatial origin of the rhythm in the BLA confirmed by comparing data from multiple recording sites (Supporting Information Fig. S1). BLA gamma frequency oscillations had mean power of 166 ± 29 μV^2^/Hz and mean frequency of 35 ± 1 Hz (*n* = 59; Fig. 1). In all experiments the gamma oscillations were highly stable over time (Fig. 1B), and showed a clear, narrow spectral peak indicative of a high degree of local coherence (Fig. 1C).

### BLA rhythms are GABA_A_ receptor dependent

**FIG. 2:**
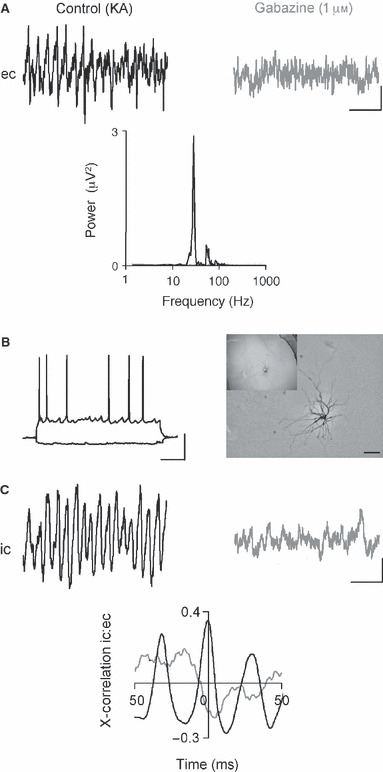
GABA_A_ receptor-mediated IPSPs are critical for oscillatory activity in BLA networks. (A) Example extracellular (ec) traces of field gamma oscillation in the BLA in control conditions and in the presence of gabazine (1 μm). The corresponding power spectra, control (black) and gabazine (grey), demonstrates the sensitivity of the BLA gamma oscillation to antagonism of the GABA_A_ receptor. KA, kainic acid. (B) At resting membrane potential, the BLA principal neuron was identified via responses to hyperpolarising and depolarising voltage steps. The cell was filled in recording to illustrate its location in the BLA. Scale bar: 100 μm. (C) Intracellular recording (ic) of cell with membrane potential held at −20 mV to reveal the IPSPs the cell is receiving during kainite-induced gamma oscillation and in the presence of gabazine. Cross-correlogram demonstrated the degree of correlation between extracellular and intracellular (principal cell IPSPs) oscillatory activity in the control gamma oscillation (black) and in the presence of gabazine (grey). Scale bars: 50 μV, 100 ms (A); 20 mV, 100 ms (B); 2 mV, 100 ms (C).

In common with population gamma rhythms observed in other cortical structures (Whittington *et al.*, 2000; Cunningham *et al.*, 2003, 2004b), oscillatory activity in the BLA was found to be critically dependent on fast inhibitory synaptic transmission mediated by GABA_A_ receptors. Application of GABA_A_ receptor antagonist gabazine (1–2 μm) abolished all oscillatory activity (Fig. 2A; control vs. gabazine; peak amplitude, 26 ± 6 vs. 1 ± 0.8 μV^2^/Hz, Student’s *t*-test, *P* < 0.05, *n* = 5). On continued gabazine application, epileptiform activity was also seen (not shown). Intracellular recordings from principal cells (Fig. 2B; *n* = 5) in the BLA revealed trains of fast IPSPs at the same frequency as the concurrently recorded local field potential and tightly phase-locked to the local population rhythm (Fig. 2C). IPSPs had a mean amplitude of 2.00 ± 0.04 mV and monoexponential decay constant of 8.0 ± 0.2 ms. These IPSPs were also abolished by application of gabazine alongside the correlation between the field and individual cell activity (Fig. 2C).

**FIG. 3:**
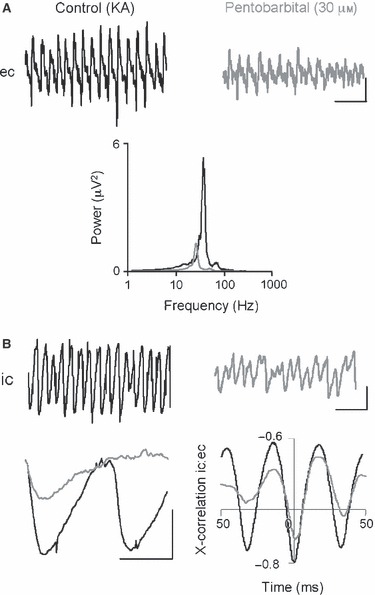
Pentobarbital reduces the frequency and amplitude of BLA gamma oscillations by modulation of BLA principal neuron IPSPs. (A) Example traces of extracellular recording (ec) of field gamma oscillation in the BLA in control gamma oscillation and in the presence of the barbituate, pentobarbital. The corresponding power spectra, control (black) and pentobarbital (grey), demonstrate the sensitivity of the BLA gamma oscillation to prolongation of the GABA_A_ receptor kinetics. KA, kainic acid. (B) Intracellular recording of cell with membrane potential held at −20 mV to reveal the IPSPs the cell is receiving in control and pentobarbital. Example traces illustrate the reduction in amplitude and increase in decay time of gamma frequency BLA principal neuron IPSPs produced by pentobarbital. Cross-correlogram [field (black) vs. principal cell IPSPs (grey)] to show the correlation between oscillatory activity and the inhibitory potentials the cell is receiving in control and pentobarbital. Scale bars: 100 μV, 100 ms (A); 5 mV, 100 ms (B); 10 mV, 20 ms (B).

Further demonstration of the importance of phasic GABA_A_ receptor-mediated inhibition was obtained by modulating GABA decay constant with pentobarbital (30 μm; e.g. Whittington *et al.*, 1995). A reduction in both the frequency and power of field gamma oscillations in the BLA was seen (Fig. 3A; control vs. pentobarbital; frequency, 34 ± 1 vs. 30 ± 1 Hz, Student’s *t*-test, *P* < 0.05, *n* = 13; peak amplitude, 33 ± 11 vs. 8 ± 3, Mann–Whitney *U*-test, *P* < 0.05, *n* = 13). Moreover, in intracellular principal cell recordings IPSP amplitudes were also reduced by pentobarbital and longer IPSP decay times (control vs. pentobarbital; amplitude, 7 ± 0.5 vs. 4 ± 0.2 mV, Student’s *t*-test, *P ≤* 0.05, *n* = 4; decay time, 14 ± 0.4 vs. 17 ± 0.5 ms, Student’s *t*-test, *P ≤* 0.05, *n* = 3), along with rhythmicity, evident from side peaks in the cross-correlation between field and individual cell IPSPs (Fig. 3B).

### The role of glutamatergic transmission for BLA gamma oscillations

**FIG. 4:**
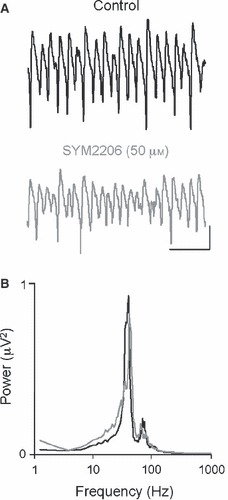
AMPA receptors have no critical role in gamma oscillations in the BLA. Application of the AMPA receptor-specific antagonist SYM 2206 (50 μm) had no significant effect on gamma oscillations in the BLA. (A) Example extracellular traces of gamma frequency oscillations induced by kainate and in the presence of the AMPA receptor antagonist, SYM 2206. (B) Corresponding pooled power spectra showing a lack of alteration in BLA gamma oscillation (black) power and frequency following AMPA receptor blockade (grey). Scale bars: 200 μV, 100 ms (A).

Phasic AMPA receptor-mediated excitation of interneurons is a fundamental feature of persistent gamma rhythms in the hippocampus (Whittington *et al.*, 2000), entorhinal cortex (Cunningham *et al.*, 2003) and neocortex (Cunningham *et al.*, 2004b). However, the AMPA receptor-specific antagonist SYM 2206 (50 μm) had no significant effect on oscillation frequency or power in the BLA (Fig. 4A and B; control vs. SYM 2206, frequency, 36 ± 1 vs. 37 ± 1 Hz, peak amplitude, 285 ± 54 vs. 248 ± 63 μV^2^/Hz, Student’s *t*-test, *P* > 0.05, *n* = 8).

**FIG. 5:**
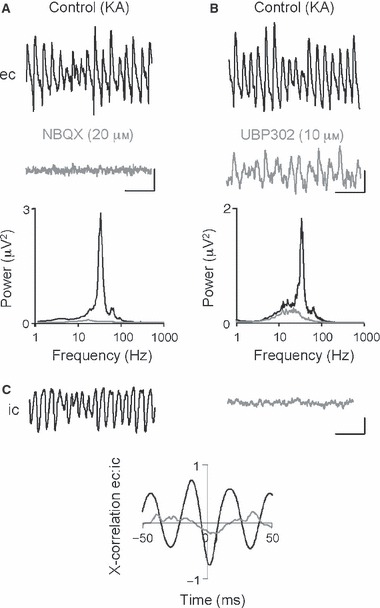
BLA gamma oscillations are sensitive to AMPA/kainate receptor antagonism. (A) Example extracellular field (ec) traces illustrating control BLA gamma oscillations and the abolition of this activity in the presence of NBQX (20 μm) and corresponding pooled power spectra. KA, kainic acid. (B) Example extracellular field (ec) traces illustrating control BLA gamma oscillations and the abolition of this activity in the presence of UBP302 (25 μm) and corresponding pooled power spectra. (C) Sample traces of concurrent intracellular IPSPs (ic) during BLA gamma oscillation and cross-correlation analysis of ec and ic recordings that demonstrates that correlation between ec and ic activity is abolished with NBQX. Scale bars: 200 μV, 100 ms (A and B); 2 mV and 100 ms (C).

**FIG. 6:**
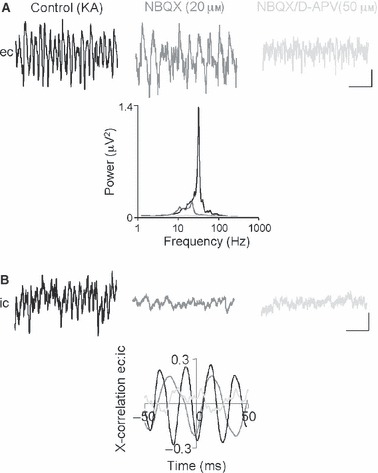
AMPA/kainate receptor antagonism can in some cases reveal a residual NMDA receptor-dependent beta oscillation. (A) Example extracellular field traces (ec) illustrate the emergence of BLA beta oscillatory activity in the presence of NBQX, and subsequent abolition of beta oscillation with d-APV. Corresponding pooled power spectra for these experiments. KA, kainic acid. (B) Sample traces of concurrent intracellular IPSPs (ic) in all experimental conditions. Associated cross-correlation analysis of ec and ic recordings demonstrates the reduction (NBQX, beta oscillation) and abolition (d-APV) between BLA ec and ic oscillations. Scale bars: 200 μV, 100 ms (A); 2 mV, 100 ms (B).

In the majority of BLA slices tested (8/14), the broad spectrum AMPA/kainate receptor antagonist NBQX abolished both field gamma rhythm generation and gamma frequency trains of IPSPs (Fig. 5A and C). GluR5-containing kainate receptor antagonist UBP302 also abolished BLA oscillations (Fig. 5B; control vs. UBP302; 24 ± 10 vs. 3 ± 1 μV^2^/Hz, Student’s *t*-test, *P* < 0.05, *n* = 4). However, in 6/14 slices exposed to NBQX, a small-amplitude, beta frequency oscillation remained in the BLA. Modal peak frequency in the power spectrum changed from a control value of 35 ± 2 Hz, down to 17 ± 2 Hz (Student’s *t*-test, *P* < 0.05, *n* = 6). When manifest, the beta oscillation was abolished by further application of the *N*-methyl-d-aspartate (NMDA) receptor antagonist D-APV (50 μm;Fig. 6A; control vs. NBQX vs. NBQX/D-APV; frequency, 35 ± 2 vs. 17 ± 2 vs. 24 ± 5 Hz, Mann–Whitney *U*-test, *P* < 0.05 for control vs. NBQX, Mann–Whitney *U*-test, *P* > 0.05 for NBQX vs. NBQX/D-APV, peak amplitude, 129 ± 27 vs. 68 ± 21 vs. 6 ± 3 μV^2^/Hz, Mann–Whitney *U*-test, *P* < 0.05 *n* = 6/14 of total slices treated with NBQX). Figure 6C shows that IPSP frequency slows correspondingly with emergence of the beta rhythm, and the IPSPs seen were both smaller (control vs. NBQX; 7.5 ± 0.4 vs. 4.5 ± 0.2 mV, Student’s *t*-test, *P* < 0.05) and had slower decay constants (control vs. NBQX; 7.1 ± 0.3 vs. 12.8 ± 0.5 ms, Student’s *t*-test, *P* < 0.05). These IPSPs were abolished alongside the residual beta rhythm on application of D-APV. Correlation between field and IPSPs alters as the oscillation frequency changes and disappears when the oscillation is abolished (Fig. 6C).

### NMDA receptor activity is not required for BLA gamma oscillations

**FIG. 7:**
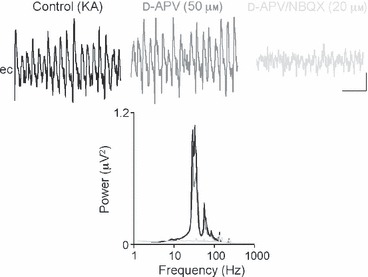
NMDA receptor blockade alone has no effect on gamma oscillations in the BLA. Sample extracellular traces show the lack of sensitivity of BLA gamma oscillations to NMDA receptor antagonism and subsequent reduction by NBQX. These data are summarised in the corresponding pooled power spectra for the three conditions control (black), d-APV (grey) and NBQX (light grey). Scale bars: 200 μV, 100 ms (A). KA, kainic acid.

In the hippocampus, NMDA receptor blockade has no significant effect on persistent gamma rhythms (Fisahn *et al.*, 1998). However, it decimates gamma power in the entorhinal cortex (Cunningham *et al.*, 2003). The effects of NMDA receptor blockade in the BLA were, again, different to that seen in the entorhinal cortex but the same as that seen in the hippocampus. D-APV (50 μm) caused no significant decrease in the frequency or peak amplitude of the local rhythm, but following NMDA receptor blockade, NBQX (20 μm) application abolished all oscillatory activity, as shown in Fig. 7A (control vs. D-APV vs. D-APV/NBQX; frequency, 35 ± 2 vs. 34 ± 2 vs. 27 ± 6 Hz, peak amplitude, 145 ± 60 vs. 195 ± 73 vs. 7 ± 2 μV^2^/Hz, *P* > 0.05 for control vs. NBQX, *P* < 0.05 for NBQX vs. NBQX/D-APV, in both cases Mann–Whitney *U*-test, *P* > 0.05, *n* = 5).

### BLA oscillations require active gap junctions

**FIG. 8:**
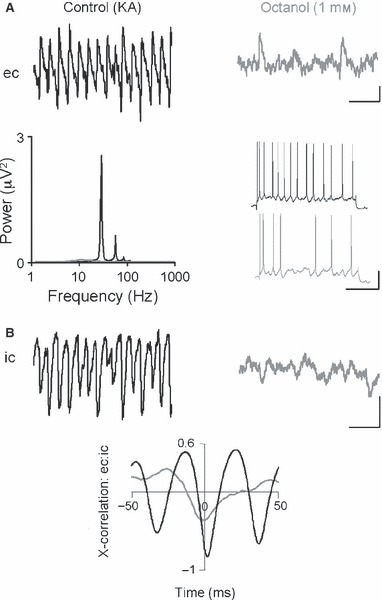
Gap junctions are required for persistent gamma oscillations in the BLA. (A) Example traces of extracellular field recording (ec) of gamma oscillation in the BLA in control and in the presence of the broad gap junction blocker, octanol (1 mm). Corresponding pooled power spectra illustrate the abolition of BLA gamma oscillation by gap junction antagonism. Example traces from BLA principal neurons in response to depolarising (+0.4 nA) steps in the absence and presence of octanol demonstrate a lack of effect on cellular excitability by the gap junction blocker. KA, kainic acid. (B) Sample traces of concurrent intracellular IPSPs (ic) in the presence and absence of the gap junction blocker. Associated cross-correlation analysis of ec and ic recordings demonstrates the disruption of synchrony between BLA ec and ic oscillations in the presence of octanol. Scale bars: 100 μV, 100 ms; 10 mV, 200 ms (A); 10 mV, 100 ms (B).

In common with previous studies in the entorhinal cortex (Cunningham *et al.*, 2004a) and the hippocampus (LeBeau *et al.*, 2003), electrical communication via gap junctions appeared vital for persistent kainate-driven oscillations in the BLA. Because of problems associated with gap junction blocker specificity, a range of gap junction blockers were applied to BLA oscillations. Bath-application of 1 mm octanol almost completely blocked the KA-induced field potential rhythm (Fig. 8A; control vs. octanol peak amplitude; 148 ± 47 vs. 9 ± 7 μV^2^/Hz, Student’s *t*-test, *P* < 0.05, *n* = 6). Similarly, the gamma frequency trains of IPSPs in principal cells, and corresponding IPSP–field potential correlates were also abolished with octanol (Fig. 8B). Furthermore, no detrimental effects of octanol were apparent in intracellular recordings (Fig. 8A), and no significant changes to cell membrane properties were observed. The resting membrane potential, input resistance and action potential amplitude of BLA principal neurons were not significantly altered by the application of octanol (control vs. octanol; resting membrane potential: 58 ± 3 vs. 58 ± 2 mV, input resistance: 27 ± 6 vs. 28 ± 6 MΩ, action potential amplitude: 49 ± 1 vs. 50 ± 1, Student’s *t*-test, *P ≥* 0.05, *n* = 4). Current injection (0.1 nA) in both the control and octanol condition was consistently able to produce similar patterns of firing in BLA principal neurons. Bath-application of other gap junction blockers, quinine (500 μm) and carbenoxolone (200 μm), also abolished gamma oscillations in the BLA (control vs. quinine; 64 ± 7 vs. 14 ± 6 μV^2^/Hz, Mann–Whitney *U*-test, *P* < 0.05, *n* = 5; control vs. carbenoxolone; 91 ± 30 vs. 18 ± 8 μV^2^/Hz, Student’s *t*-test, *P* < 0.05, *n* = 8).

## Discussion

The present data demonstrate that the BLA generates gamma frequency population rhythms when activated by KA. Whilst previous reports have observed spontaneous gamma frequency oscillations in the hippocampus (Pietersen *et al.*, 2009), such activity was not observed in the BLA in this current study. The gamma rhythm generated was near-identical to that seen for gamma rhythms in area CA3 (30–40 Hz), but slower than that reported for area CA1 in the hippocampus (40–100 Hz; Middleton *et al.*, 2008a, b). BLA gamma rhythms were absolutely dependent on GABA_A_ receptor-mediated inhibition, as seen for all other persistent gamma rhythm models *in vitro*. Blockade of GABA_A_ receptors abolished the rhythms, and slowing of GABA_A_ time constants with barbiturates also slowed the population rhythm. However, in contrast to these rhythms in the hippocampus, entorhinal and neocortical structures, BLA gamma rhythms did not require phasic AMPA receptor-mediated excitation at all, but did depend on GluR5-containing kainate receptors. In addition, a low-amplitude NMDA receptor-dependent beta frequency oscillation was also seen when gamma rhythms were blocked.

The precise nature of the frequency of the BLA rhythm may be of critical importance in shaping its involvement in the amygdala–hippocampal–entorhinal axis, as has been shown to occur in learning paradigms (Bauer *et al.*, 2007). Coupling and decoupling along this axis has been implicated in specific types of memory (Kensinger & Schacter, 2006). It is interesting to note that *in vitro* studies have shown a large discrepancy in gamma frequencies generated internally by hippocampal area CA3 (about 35 Hz) and area CA1 and entorhinal cortex (about 45–55 Hz). The peak, persistent gamma frequency seen in the BLA appeared to match CA3 hippocampal gamma frequencies, but not those generated in area CA1 or entorhinal cortex. The residual beta rhythm seen in the BLA may also be critical for controlling dynamic interactions between amygdala and hippocampus. Beta rhythms are seen during serotoninergic neuromodulation in the hippocampus (Bibbig *et al.*, 2007) and, in general, are thought to provide a temporal framework more suitable for generating synchrony over larger spatial scales than gamma rhythms alone (Kopell *et al.*, 2000).

The profile of AMPA/KA receptor involvement in the BLA rhythms may, in part, be due to the differences in receptor subunit composition in the BLA compared with the hippocampus and entorhinal cortex, but this was not explored here. AMPA receptor blockade with SYM 2206 had no significant effect on KA-driven BLA oscillations, whereas AMPA receptors played a large role in hippocampal (Whittington *et al.*, 2000) and entorhinal oscillations (Cunningham *et al.*, 2004a). Moreover, the specific KA receptor antagonist UBP302 abolished oscillations, suggesting GluR5-containing KA receptors provided the predominant fast, excitatory drive to BLA gamma oscillations. Evidence in the literature supports a mechanistic role for GluR5-containing kainate receptors in BLA functions, and potentially including oscillatory activity generation. The BLA contains a high density of GluR5-containing KA receptors (Wisden & Seeburg, 1993; Li *et al.*, 2001), shown to be important for some forms of amygdala-dependent learning (Ko *et al.*, 2005). Their activation was shown to decrease GABA release, reducing overall IPSP amplitude and generating an increased excitability in the local field potential (Braga *et al.*, 2003). It has previously been shown that GluR5-containing KA receptors modulate GABAergic inhibition in the BLA (Wu *et al.*, 2007). Removal of GluR5-containing KA receptor activity in GluR5 knockout mice or with infusion of a GluR5 antagonist increases anxiety-like behaviour, and this occurs as GABA release is reduced when inhibitory circuits have no GluR5 activation to stimulate GABA release. Overall this results in amygdala activation via loss of inhibition (Wu *et al.*, 2007), potentially a loss of inhibitory tone in the form of oscillations. These receptors also have novel roles in synaptic potentiation in the BLA, not reported in other brain regions, which involves numerous synapses and occurs after low-frequency stimulation (Li *et al.*, 2001). It is also NMDA receptor independent, as opposed to Hebbian synaptic mechanisms that are input and NMDA receptor dependent (Li *et al.*, 2001). It is possible that GluR5-containing kainate receptors influence behaviours through their effects on BLA oscillations. However, it must be noted that although AMPA receptor blockade does not affect the oscillations, application of kainate receptor-specific agonist ATPA only generated small levels of network activity. This implies that AMPA-mediated drive may contribute to rhythm generation rather than maintenance.

While the present data show a specific role for NMDA receptors in the BLA beta – but not gamma – rhythm, there is also evidence to suggest that different interneuron subpopulations may be involved in this type of activity. Although not directly related to this study, and in mice, Marowsky *et al.* (2005) identified the subpopulations of interneurons and showed that the firing properties of local interneurons, which are scattered throughout the BLA, had a peak firing frequency within the gamma range. In contrast, lateral and medial paracapsular cells, which are located between the lateral BLA border and external capsule and between the medial BLA border and central nucleus, respectively, had peak firing frequencies in the beta frequency range. Different BLA rhythms could be features of independently acting networks within the BLA. In accordance with this theory, the difference in amplitude and decay kinetics for IPSPs accompanying gamma and beta rhythms seen in the present data strongly suggest different origins for the phasic inhibition underlying each rhythm; however, no interneuron recordings were made in this study and this is an aim for the continuation of this work. Evidence of a differential topography of GABAergic inhibition onto principal neurons is known from work in the neocortex (Tamás *et al.*, 2004), and a similar divergence of inhibition may underlie the generation of gamma and beta rhythms in the BLA.

Gamma rhythms in the BLA shared the sensitivity to gap junction blockers seen in the hippocampus and entorhinal cortex (Cunningham *et al.*, 2003; LeBeau *et al.*, 2003). Neurons in the BLA are highly interconnected via gap junctions, and anatomical studies have shown that interneurons, in particular, are interconnected by both dendritic and axo-axonic gap junctions at terminals (Muller *et al.*, 2005). Dendritic gap junctions are present in hippocampal interneuron networks, and their removal disrupts gamma oscillations (Hormuzdi *et al.*, 2001), and this could also explain the gap junction requirement for BLA oscillations. So, although the BLA lacks a laminar composition that is thought to contribute to the axonal plexus-mediating drive to oscillations (Traub *et al.*, 2003), there is a structural organisation that acts like the axonal plexus in enhancing electrical communication within networks (Traub *et al.*, 2008). Oscillatory activity in the LA was previously shown to be gap junction dependent (Sinfield & Collins, 2006).

The present data showed that BLA networks in isolation can generate gamma frequency oscillations *in vitro*. BLA network oscillations *in vivo* are synchronised with other regions during common tasks (e.g. Popescu *et al.*, 2009). The similarity in frequency bands generated in BLA (gamma and beta seen here, and theta and gamma reported in different conditions by Sinfield & Collins, 2006) strongly suggests that this synchrony may be afforded by dynamic interactions between locally generated rhythms in BLA and these coupled regions. The network mechanisms responsible for these rhythms in BLA were similar, but not identical, to those in other cortical areas studied. The complex, non-laminar structure of the amygdala makes it less amenable for *in vitro* investigation than laminar cortical areas. However, this *in vitro* model of amygdala oscillations may provide a platform for further investigation into the mechanisms of oscillation generation in the BLA, and thus its role in modulating and coordinating higher brain function.

## Abbreviations

aCSF: artificial cerebrospinal fluid
AMPA: α-amino-3-hydroxy-5-methyl-4-isoxazolepropionic acid
ATPA: (*RS*)-2-amino-3-(3-hydroxy-5-*tert*-butylisoxazol-4-yl)
BA: basolateral nuclei
BLA: basolateral amygdala
D-APV: D-(−)-2-amino-5-phosphonovalerate
GABA: γ-aminobutyric acid
IPSP: inhibitory postsynaptic potential
KA: kainic acid
LA: lateral nuclei
NBQX: 6-nitro-7-sulphamoylbenzo[f]quinoxalone-2,3-dione disodium
NMDA: *N*-methyl-d-aspartate
SYM 2206: (±)-4-(4-aminophenyl)-1,2-dihydro-1-methyl-2-propylcarbamoyl-6,7-methylenedioxyphthalazine
UBP302: (*S*)-1-(2-amino-2-carboxyethyl)-3-(2-carboxybenzyl) pyrimidine-2,4-dione

## Supporting Information

**Fig. S1.** Profile of gamma frequency oscillationin BLA *in vitro*. (A) Slice diagram shows the slicecomposition and the numbered recording position. As alreadydiscussed, lack of laminar structure in BLA prevents identificationof the exact recording site, and so this method was used to showthat oscillatory activity within the slice was localised to the BLAregion. (B) Corresponding power spectra for each position denotedin (A).

Please note: As a service to our authors and readers, this journal provides supporting information supplied by the authors. Such materials are peer-reviewed and may be re-organized for online delivery, but are not copy-edited or typeset by Wiley-Blackwell. Technical support issues arising from supporting information (other than missing files) should be addressed to the authors.
