# Supplementary figures and images for: Fast oscillatory activity induced by kainate receptor activation in the rat basolateral amygdala in vitro
Source: Eur J Neurosci. 2011 Mar;33(5):914–22. doi: 10.1111/j.1460-9568.2010.07582.x (PMC3627304; doi:10.1111/j.1460-9568.2010.07582.x)

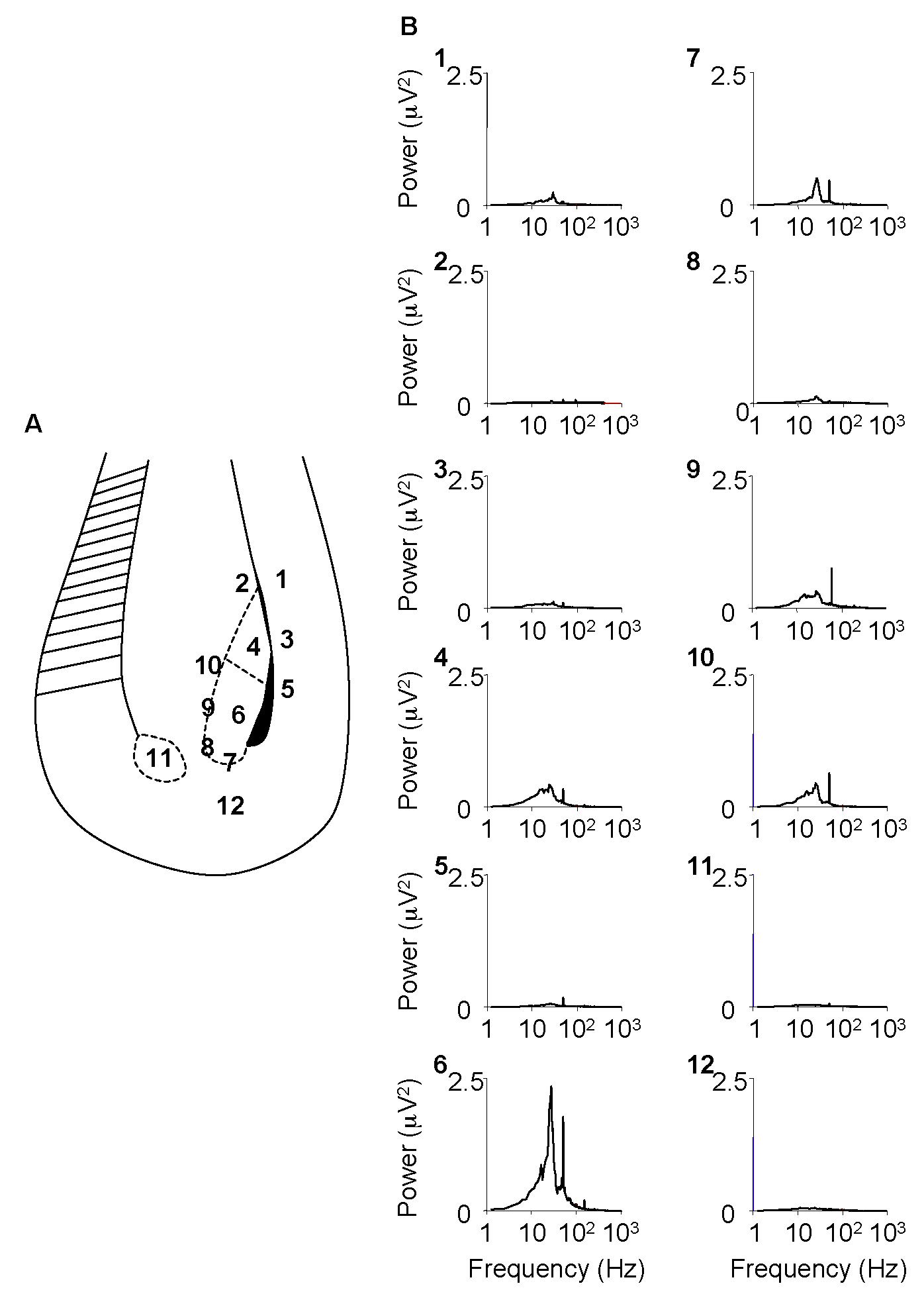

Supplement: Supplementary file 1 [file ejn0033-0914-SD1.tiff]
